# E-cigarettes with vitamins and nutrients: where quackery and technology meet

**DOI:** 10.1590/0102-311XEN024223

**Published:** 2024-02-02

**Authors:** Andre Luiz Oliveira da Silva

**Affiliations:** 1 Center for Tobacco Control Research and Education, University of California, San Francisco, U.S.A.

Electronic devices to smoke (DEF, acronym in Portuguese) are equipment that aim to simulate the act of tobacco smoking. DEF can be classified according to their matrix: solid, liquid, or hybrid (Figure 1). As an example, we can mention electronic cigarettes (e-cigarettes), which consist of a liquid matrix, and heated tobacco products that use a solid matrix.

**Figure 1:**
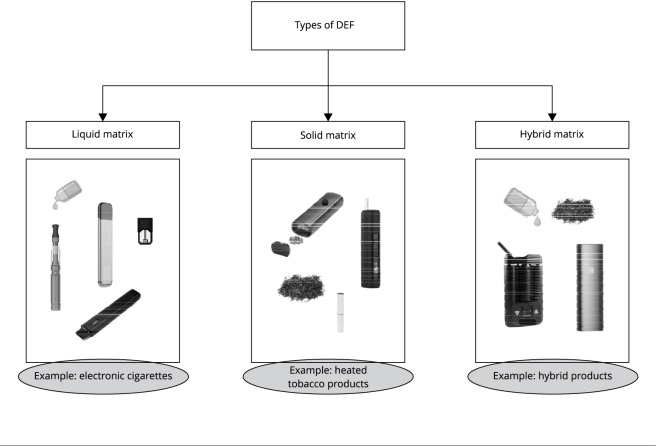
Types of electronic devices to smoke (DEF) according to the nature of the matrix used.

Source: prepared by the author.

Some brands of DEF have achieved great popularity among young people, especially because of their attractive yet discreet design, technological appeal, high concentrations of nicotine salts, attractive flavors, and aggressive marketing ^1^.

These products are banned in Brazil through the Brazilian Health Regulatory Agency’s (Anvisa) *Board Resolution n. 46/2009* ^2^, which could explain the low prevalence of use when compared to countries that have authorized their commercialization ^3^. According to the *Brazilian National Health Survey* (PNS 2019, individuals ≥ 15 years old) ^4^ the prevalence of use of DEF is 0.64%. In addition, a study ^5^ indicates that dual use is 10 times higher in the 18-24 age group when compared to the 35+ age group; half of those who have used DEF in their lifetime have never smoked, 80% of them are aged 18-34; and a large portion had a high level of education. For these reasons, some authors consider DEF to be a threat to tobacco control in Brazil ^5^. In this scenario, at the end of January 2023, an advertisement for an e-cigarette branded IZ Health, composed of vitamins and other nutrients, circulated on social networks. The company advertisement claimed that these vitamins would be absorbed by the mucous membranes. This advertisement raised a number of discussions and criticisms, especially from health professionals ^6^. In the video, a young athlete in a gym reports using e-cigarettes to boost one’s vigor and energy to perform exercises and daily tasks.

## About the product 

According to photos and internet searches, the product called “Power”, would consist of vitamin B_12_, l-carvone, l-theanine, and caffeine. The packaging provided no information about the manufacturer or importer. The person responsible for advertising the product stated that it did not contain nicotine nor tobacco.

The research indicated that the person responsible for the product apparently put their logo and colors on a product manufactured by the U.S. company Health Vape. These products were only found on online stores aimed at the Brazilian public, so we can assume that those responsible for the product were Brazilian, despite the fact that the images in the advertisements were in English.

The investigation also revealed that in addition to the model indicated to increase physical vigor, there were also formulations indicated for rejuvenation, relaxation, immune support, sleep improvement, and maintenance of focus. Box 1 shows models, declared compositions, and manufacturer’s indications. The declared compositions included vitamins, amino acids, collagen, and plant extracts.

**Box 1:** Models, manufacturer indications, and comments on IzHealth brand e-cigarettes with vitamins advertised on the Internet.

MODEL	MANUFACTURER’S INDICATION	DECLARED COMPOSITION	COMMENTS
Restore	Rejuvenation	Collagen	The efficacy of oral collagen supplementation for aesthetic improvement of the skin is inconclusive, especially considering that the protein is not fully absorbed under normal conditions ^20^. There are no tests of safety, efficacy, or proof of properties via inhalation.
		Geranium	Popularly used for skin lesions, there is no evidence that it provides benefits regarding skin aging ^21^. There are no tests of safety, efficacy, or proof of properties via inhalation.
		l-carnitine	Indicated in sports for improving performance, but the scientific evidence is inconsistent. There are no data on the ability of this molecule to delay aging ^22^. There are no tests of safety, efficacy, or proof of properties via inhalation.
		Glutathione	No solid scientific evidence supports the anti-aging claims ^23^. There are no tests of safety, efficacy, or proof of properties via inhalation.
		β-Ionone	Precursor of vitamin A. There is no evidence that dietary supplementation of β-Ionone in healthy individuals provides benefits for the skin and hair ^24^. There are no tests of safety, efficacy, or proof of properties via inhalation.
Vital	Immune support	Coenzyme Q_10_	There is no evidence that oral supplementation in healthy individuals improves immune system performance ^25^. There are no tests of safety, efficacy, or proof of properties via inhalation.
		Vitamin C	There is no evidence that oral supplementation in healthy individuals improves immune system performance ^26^. There are no tests of safety, efficacy, or proof of properties via inhalation.
		Vitamin D_3_	There is no evidence that oral supplementation in healthy individuals improves immune system performance ^27^. There are no tests of safety, efficacy, or proof of properties via inhalation.
		Vitamin B_12_	Data suggest the possibility of its use to treat viral infections ^28^. Intranasal administration may be more efficient than oral administration, but sublingual absorption is less efficient than oral absorption. There are no long-term safety studies ^29^.
		Vitamin A	There is no evidence that oral supplementation in healthy individuals improves immune system performance ^30^. There are no tests of safety, efficacy, or proof of properties via inhalation.
Zen	Relaxation	Chamomile	Chamomile is used orally as a sedative ^31^. There are no tests of safety, efficacy, or proof of properties via inhalation.
		Passiflora	Traditionally known for oral use as a sedative. Clinical studies partially confirm its activity, but limitations in these studies prevent its registration as a medicine ^32^. There are no tests of safety, efficacy, or proof of properties via inhalation.
		Valerian root	Oral use as a sedative is well established ^32^. There are no tests of safety, efficacy, or proof of properties via inhalation.
		l-theanine	The claimed stress-reducing and sleep-improving properties hold no scientific evidence to back them up ^33^. There are no tests of safety, efficacy, or proof of properties via inhalation.
Melatonin	Rest	Melatonin	Studies suggest some possible improvement in specific conditions such as jetlag and shift workers. The evidence for consuming this substance to combat insomnia is inconclusive, and no evidence has been found of an improvement in the quality of sleep in healthy people ^34^. There are no tests of safety, efficacy, or proof of properties via inhalation.
		Passiflora extract	Traditionally known for oral use as a sedative. Clinical studies partially confirm its activity, but limitations in these studies prevent its registration as a medicine ^32^. There are no tests of safety, efficacy, or proof of properties via inhalation.
		Chamomile	Chamomile is used orally as a sedative and for gastrointestinal problems ^31^. There are no tests of safety, efficacy, or proof of properties via inhalation.
		Valerian root	Oral use as a sedative is well established ^32^. There are no tests of safety, efficacy, or proof of properties via inhalation.
		l-theanine	The claimed stress-reducing and sleep-improving properties hold no scientific evidence to back them up ^33^. There are no tests of safety, efficacy, or proof of properties via inhalation.
Power	Energy	Vitamin B_12_	Data suggest the possibility of its use to treat viral infections ^28^. Intranasal administration may be more efficient than oral administration, but sublingual absorption is less efficient than oral absorption. There are no long-term safety studies ^29^. No evidence of improved activity levels or disposition has been described. Intranasal administration may be more efficient than oral administration, but there are no long-term safety studies and some concerns about mucosal damage. Sublingual administration; and less absorbed than the oral route.
		l-carvone	Animal studies suggest a possible sedative effect ^35^. There are no tests of safety, efficacy, or proof of properties via inhalation.
		l-theanine	The properties claimed of improving cognitive function are unproven ^33^. There are no tests of safety, efficacy, or proof of properties via inhalation.
		Caffeine	There is consistent evidence of reduced pain perception and exertion. Possibly improves performance in endurance activities and long-duration intermittent activities ^36^. There are no tests of safety, efficacy, or proof of properties via inhalation.
Boost	Focus	Vitamin B_12_	Data suggest the possibility of its use to treat viral infections ^9^. Intranasal administration may be more efficient than oral administration, but sublingual absorption is less efficient than oral absorption. There are no long-term safety studies ^29^. No evidence of improved activity levels or disposition has been described. Intranasal administration may be more efficient than oral administration, but there are no long-term safety studies and some concerns about mucosal damage. Sublingual administration; and less absorbed than the oral route.
		Vitamin B_6_	Involved in cognitive development, biosynthesis of neurotransmitters, among other functions ^37^. No evidence of improved activity levels or disposition has been described. There are no tests of safety, efficacy, or proof of properties via inhalation.
		l-theanine	The properties claimed of improving cognitive function are unproven ^33^. There are no tests of safety, efficacy, or proof of properties via inhalation.
		l-lysine	Data show that it does not improve performance or improve recovery from exhaustion ^36^. No evidence of improved activity levels or disposition has been described. There are no tests of safety, efficacy, or proof of properties via inhalation.
		Taurine	Among athletes, this supplement is used to improve performance, but the evidence is limited ^38^. There are no tests of safety, efficacy, or proof of properties via inhalation.

Source: prepared by the author.

Note: there is no evidence that dietary supplementation of vitamins and minerals in healthy individuals brings any additional health benefit ^6^.

When analyzing the advertised composition of the manufacturers’ products, it is noticeable that the concentrations of the substances used are absent. On the advertised packaging, it is also not possible to identify the composition of the vehicle used in the product. In the case of Health Vape, the possible origin of the Brazilian version, the manufacturer stated that the vehicle was propylene glycol.

## Intake of nutrients via respiratory tract

When analyzing the declared components of the product (Box 1), it can be seen that some components present inconsistent data on their efficacy even when orally consumed. Excepting vitamin B_12_, the components hold no tests of absorption or equivalence via airways. Thus, these products hold no indication of benefiting their users ^7^. The promotion of DEF with nutrients is similar to that found on the U.S. market ^8^.

## About the safety of consuming these products

The advertiser of the product indicates that these products are safe and beneficial to health. However, when we looked at the components (Box 1), we found that none of the substances used have been tested for inhalation toxicity. In some cases, when the advertiser states that the consumption of these products is safe, ignores, for example, that excessive consumption of vitamin A can cause damage to health, especially considering that the concentrations are not shown.

Moreover, it is important to remember that a substance that is safe for oral consumption will not necessarily be safe for inhalation. Diacetyl (2,3-butanediene) is a good example, which is used as a flavoring in several products, including microwave popcorn and butter flavoring. However, the literature presents cases of occupational exposure to diacetyl, where people inhaled the compound and developed bronchitis obliterans, also known as “popcorn lung” ^9^. Diacetyl has also been found in some brands of e-cigarettes and could be potentially harmful to individuals who smoke them ^9^.

Another relevant case is vitamin E acetate, usually consumed as a food supplement and recommended for the treatment of vitamin E deficiency. However, this same substance is one of the main suspects of having caused the cases of EVALI (chemical pneumonia caused by the use of e-cigarettes), which caused 2,870 hospitalizations and 68 deaths (2020 data) in the United States ^10^.

Thus, the claims that a substance is safe when consumed orally cannot be used to assert that this same substance would be safe when inhaled.

## Vitamins and nutrients in smoking products

The scientific literature describes that the use of vitamins and nutrients in DEF, whether they contain nicotine or not, has been practiced since at least 2018, and in addition to the indications mentioned above, brands sold on the international market are also indicated for weight control by appetite suppression ^8^.

However, the use of vitamins in other tobacco products dates back to the 1980s-1990s, and basically began to be studied by the tobacco industry in an attempt to mitigate the health damage caused by cigarettes ^11^ ^,^ ^12^. Several substances have been tested, including β-carotene, vitamins B_1_, B_2_, C and E, provitamin A, catechin, eugenol, bioflavonoids, vanillyl, tryptophan, turmeric, glutathione, ethyl salicylate, and essential oils. Even the use of genetically modified tobacco plants with genes to produce beta carotene has been considered ^11^.

A “vitaminized” cigarette was commercialized in the Canadian market in 2006. The VitaCig, a conventional cigarette with added vitamin C would be less likely to cause stains on the teeth, hold less odor, and be healthier by guaranteeing doses of vitamin for smokers, at least according to the manufacturer ^13^.

Research aimed at evaluating the use of vitamins to mitigate the damage caused by cigarettes may originate from antioxidant effects of vitamins, which might be a possible defense against the free radicals present in tobacco smoke and from the fact that smokers have reduced levels of some vitamins ^14^. However, this idea seemed to be weakened by a study suggesting that β-carotene supplementation in smokers could increase the incidence of lung cancer and heart disease ^15^.

In the advertisement, the product is used in an enclosed environment, implying that it would not impact the people’s health. This type of attitude, in addition to being a health infraction, as discussed below, induces the population to believe that these products would not cause air quality problems for non-users. However, even in products without tobacco or nicotine, their smoke emissions are potentially harmful to health and the use of these products in enclosed public spaces should not be encouraged ^16^.

## Legislation

Considering these products only in a recreational context and as a simulacrum of a cigarette, DEF cannot be marketed in Brazil, as provided by the Anvisa in *Board Resolution n. 46/2009* ^2^. Advertising them is also prohibited.

However, this situation exemplifies the challenges of regulating advertisements on the world wide web, especially on social networks. Due to its nature and ability to target specific groups, social networks poses great challenges for tobacco control policies and points to the need to develop specific strategies and tools ^17^.

Another important point is that in the advertisement, e-cigarettes is used in a gym, an action forbidden by *Law n. 9,294/1996* ^18^, which prohibits the use of any smoking product derived or not from tobacco in enclosed collective environments.

We should also remember that the manufacturer alleges unproven therapeutic properties, which could possibly fit the *Brazilian Penal Code* ^19^, specifically Chapter III (*Crimes Against Public Health*) articles 283 and 284, which deal with quackery (inculcating or announcing a cure by secret or infallible means) and faith healing (prescribing, administering, or habitually applying any substance) .

In addition to having committed a sanitary infraction by failing to comply with Anvisa’s resolution, this product could also be subject to criminal prosecution. It could also be debated whether advertising on social networks, which is available for children to watch without any warning, could fit other legislation, such as the *Statute of the Child and Adolescent*.

It is important to mention that Anvisa’s *Board Resolution n. 46/2009* ^2^ is currently being revised, and tobacco companies have been requesting that these products be legally marketed, as these products would be a safer alternative for adult smokers of conventional cigarettes.

However, as aforementioned, the data suggest that these products are particularly appealing for younger people, those who have never smoked conventional cigarettes, and those with higher levels of education, thus posing a threat to tobacco control policies in Brazil ^5^. The fact that these products are prohibited in Brazil could explain the relatively low prevalence of use among younger people, compared to other countries ^3^. Further research would be important to assess the impacts of this type of advertising on the regulatory review process or on the consumption patterns of these products.

## Final considerations

E-cigarettes with added vitamins and other nutrients, in addition to lacking any proof of health benefits, can cause damage to health. Advertisements and claims of this type try to exploit the popular belief, not always supported by scientific evidence, that nutritional supplementation in healthy individuals would bring health benefits.

This type of products are an additional challenge for health professionals, because as well as holding technological appeal and a beautiful design, they also carry claims, not scientifically proven, of health benefits, using social networks that allow these products to be advertised and marketed without any regulation.

Therefore, information campaigns to the population, stricter enforcement actions against these manufacturers, and discussing the responsibilities of social networks would be necessary initiatives to ensure that the national tobacco control policy is preserved.
